# Differences in immune status and fecal SCFA between Indonesian stunted children and children with normal nutritional status

**DOI:** 10.1371/journal.pone.0254300

**Published:** 2021-07-29

**Authors:** Ingrid S. Surono, Fasli Jalal, Syukrini Bahri, Andreas Romulo, Pratiwi Dyah Kusumo, Erida Manalu, Yusnita, Koen Venema

**Affiliations:** 1 Faculty of Engineering, Food Technology Department, Bina Nusantara University, Jakarta, Indonesia; 2 Faculty of Medicine, Department of Nutrition, YARSI University, Jakarta, Indonesia; 3 Faculty of Medicine, Clinical Pathology Department, YARSI University, Jakarta, Indonesia; 4 Faculty of Medicine, Universitas Kristen Indonesia, Jakarta, Indonesia; 5 Centre for Healthy Eating & Food Innovation, Maastricht University—Campus Venlo, Venlo, The Netherlands; McMaster University, CANADA

## Abstract

We recently showed that the gut microbiota composition of stunted children was different from that of children with normal nutritional status. Here, we compared immune status and fecal microbial metabolite concentrations between stunted and normal children, and we correlated macronutrient intake (including energy), metabolites and immune status to microbiota composition. The results show that macronutrient intake was lower in stunted children for all components, but after correction for multiple comparison significant only for energy and fat. Only TGF-β was significantly different between stunted children and children of normal nutritional status after correction for multiple comparisons. TNF-alpha, IL-10, lipopolysaccharide binding protein in serum and secretory IgA in feces were not significantly different. Strikingly, all the individual short-chain and branched-chain fatty acids were higher in fecal samples of stunted children (significant for acetate, valerate and total SCFA). These metabolites correlated with a number of different microbial taxa, but due to extensive cross-feeding between microbes, did not show a specific pattern. However, the energy-loss due to higher excretion in stunted children of these metabolites, which can be used as substrate for the host, is striking. Several microbial taxa also correlated to the intake of macronutrients (including dietary fibre) and energy. *Eisenbergiella* positively correlated with all macronutrients, while an uncharacterized genus within the Succinivibrionaceae family negatively correlated with all macronutrients. These, and the other correlations observed, may provide indication on how to modulate the gut microbiota of stunted children such that their growth lag can be corrected. Trail registered at https://clinicaltrials.gov/ct2/show/NCT04698759.

## Introduction

Malnutrition refers to deficiencies, excesses or imbalances in energy/or nutrient intake, and covers two broad groups of conditions, ‘undernutrition’, which includes stunting, wasting, acute malnutrition, underweight and micronutrient deficiencies, and ‘overweight and obesity’ [1]. Malnutrition has become a huge global burden, especially for children in many developing countries, countries suffering from wars and refugee crises [2].

The prevalence of undernutrition is most commonly observed in the developing countries, and occurs due to malnourishment, poor quality of diet, unhygienic environment, and repeated number of infections. Approximately, 151 million (22%) children under five-years-old in 2017 were affected by stunting and mostly observed in Asia [3]. Childhood malnutrition is associated with metabolic changes later in life, during adulthood [4]. Moreover, undernutrition in children less than five years old is responsible for almost half of all deaths in this age-group [5].

According to World Health Organization, stunting represents poor linear growth during a critical period. It is diagnosed as a height for age more than −2 standard deviations from the median of the WHO reference dataset [6]. Based on this standard, Indonesia is one of the leading countries that has the highest prevalence of stunting in children under-five (36.4%) [7]. It became the fifth highest among the developing countries that have the burden of stunting cases [8]. Stunting has negative impacts on early childhood development, and could disturb the full potential height during a child’s growing period, result in poor cognitive and motoric development, eventually leading to the lack of intellectual capacity and educational performance [9].

Stunting in recent studies has been linked with the human gut microbiota and immune system [10]. The human gut is colonized by numerous microorganisms, which are largely commensal and play an important role in the development of the host immune system and many physiological functions for survival of the host [11]. The gut microbiota contributes to intestinal epithelial cell proliferation and maturation, the induction of host genes for nutrient uptake and the development of the mucosal immune system, all of which are critical to optimal nutrient absorption. The gut microbiota is able to form short-chain fatty acids (SCFA) and vitamins (B3, B5, B6, B12, K, biotin and tetrahydrofolate), as well as to optimize mineral absorption [12]. These substances are pivotal for metabolic processes required to achieve an optimum level of physical growth and development. We recently showed that the gut microbiota composition of stunted children was different from that of children with normal nutritional status [13].

Such an imbalance in the intestinal microbiota (dysbiosis) may alter the functions of innate and adaptive intestinal immunity. Less diverse composition or immature microbiota is attributed to a lower absorption of consumed nutrition, which causes imbalance of innate immunity. Moreover, in stunted children, the immune system is underdeveloped. This renders these children vulnerable upon initial and recurrent infections. Furthermore, an unresolved disrupted immune system leads to hormonal nuisance and growth suppression.

Bacterial fermentation products, particularly short-chain fatty acids (SCFAs) including acetate (C2), propionate (C3), and butyrate (C4), mediate the effects on host physiology and immunity, regulating the function and differentiation of virtually all immune cell gut [14,15].

This study aimed to explore the interrelationships between the gut microbiota profile, immune response status and stool SCFA and branched-chain fatty acids (BCFA) concentrations of stunted Indonesian children and their age-matched counterpart of normal nutritional status.

## Materials and methods

### Subjects

**Fig 1:**
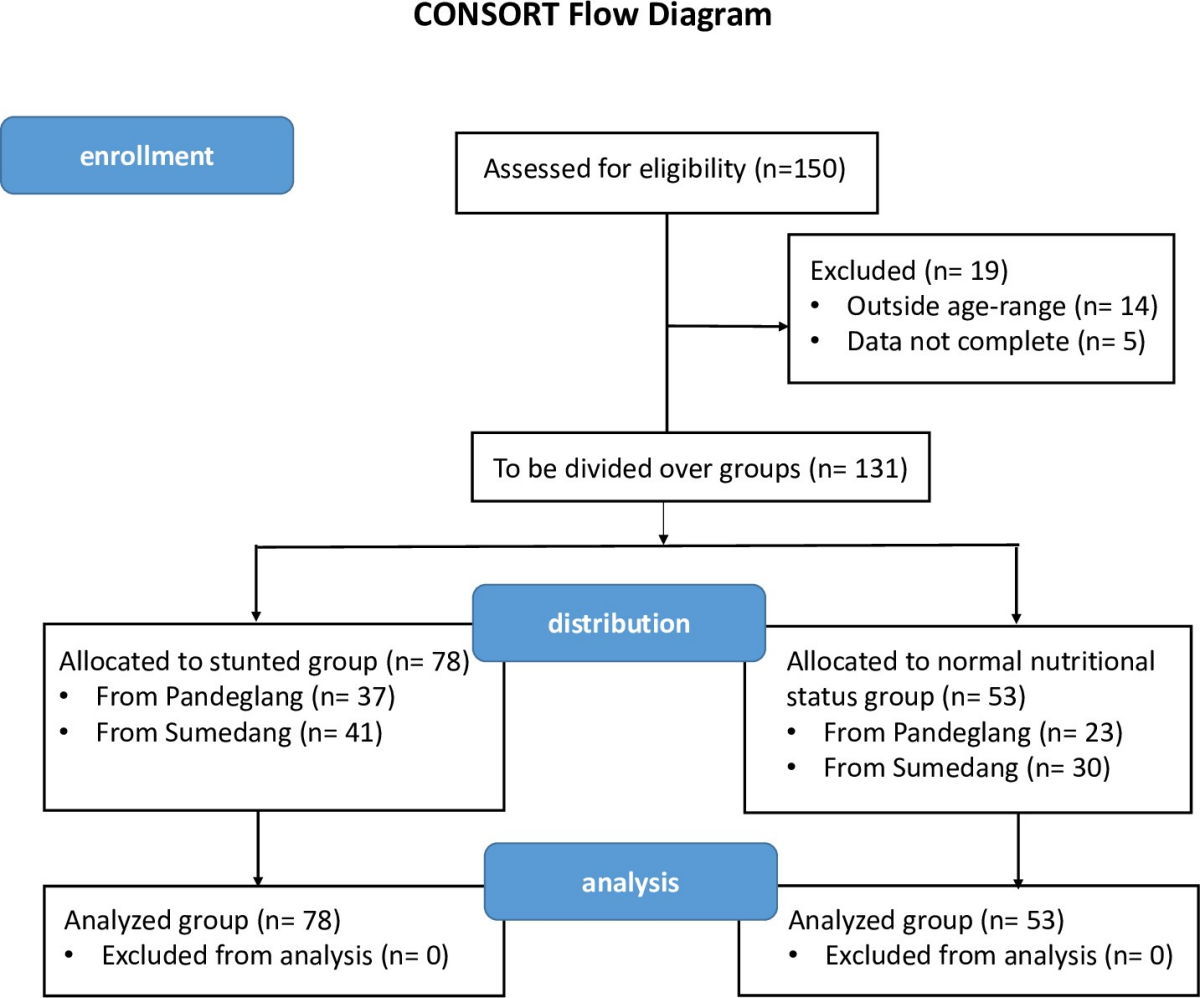
CONSORT flow diagram of the cross-sectional study.

A cross-sectional study was conducted on young children aged 3–5 years, and divided into two groups, stunted (n = 78) and normal nutritional status (n = 53), from two locations, Pandeglang District, Banten province, and Sumedang District, West Java province, Indonesia (Fig 1). The protocol was approved by the Research Ethics Committee of the Medical Faculty of Yarsi University (dossier No. 004/KEP-UY/BIA/I/2020). Written informed consent was obtained from parents or guardians of the children in the presence of a third person. Anthropometric and demographic data of the subjects were also obtained. The trial was registered at ClinicalTrials.gov under number NCT04698759. The authors confirm that all ongoing and related trials for this study are registered.

### The nutritional status of children under five

Stunting anthropometric measurements are measured based on parameters of length/height according to age compared to the WHO anthropometric standards 2005 and the Indonesian Minister of Health Decree No. 1995/MENKES/SK/XII/2010.

The nutritional status of each children included in this study was quantified using the WHO recommended three nutritional Z-scores namely, height for age (referred to in this study as Z-score 1); weight for age (referred as Z-score 2) and weight for height (referred as Z-score 3).

A structured questionnaire was used for face-to-face interviews with the respective child’s mother to collect sociodemographic information. In addition, age and anthropometric measurements (height, weight) based on Department of Health Ministry of Indonesia Regulation were recorded. For stunting, the thresholds for height-for-age are: ‘severely stunted’ (<-3 SD); ‘stunted’ (-3 SD to < -2 SD); ‘normal’ (-2 SD to +3 SD); ‘tall’ (> +3 SD). Furthermore, in order to obtain an overall measure of the nutritional status of these children, the children were classified in weight-for-height categories: ‘severely wasted’ (<-3 SD); ‘wasted’ (-3 SD to < -2 SD); ‘normal’ (-2 SD to +1 SD); ‘possible risk of overweight’ (+1 SD to +2 SD); ‘over weight’ (> +2 SD to +3 SD); ‘obese’ (> +3 SD)

The dietary intake was recorded by 24 hours food-recall and calculated for energy intake using Nutrisurvey 2007 Questionnaire application (www.nutrisurvey.de).

### Blood and stool collection

Blood samples (5 ml) were collected from stunted (n = 78) and normal nutritional status (n = 53) Indonesian children from two different locations, Pandeglang, Banten province and Sumedang, West Java province, by using a 0.9-mm needle (Precision Glide; Becton-Dickinson Vacutainer systems, Plymouth, United Kingdom). Sera were obtained by centrifugation at 3,500 x g for 30 min at 4°C, and transferred to polyproylene tubes (Eppendorf Safe-Lock Tubes), and stored at—20°C until immune and LBP assessments. Stool samples were collected, without urine, from all children using stool containers. The stools were immediately transferred to a container, and stored in a cold temperature box using an ice pack while being transported to the lab.

### IL-10 measurement

Human IL-10 in serum was prepared using Human IL-10 ELISA Kit Picokine according to manufacturer’s procedures (Booster Biological Technology, Pleasanton CA). Ninety-six-well Immulon IV ELISA plates were pre-coated with specific antibody for IL-10. Serum was diluted and added to the microplate and incubated for 60 min at 37°C. Plates were washed four times, and incubated with a biotinylated anti-human IL-10 antibody. Concentration of IL-10 in samples was measured at optical density (OD) 415 nm using microplate reader (TECAN Infinite M200, TECAN, Männedorf, Switzerland).

### TGF-β measurement

The measurement of TGF β-1 on serum was conducted according to the protocol mentioned on the TGF β-1 ELISA Kit (Merck KGaA, Darmstadt, Germany). Prior to the analysis, all reagents and buffer were brought to room temperature. The standard solution of human TGF β-1 was prepared by adding 400 μL diluent buffer to prepare a 125 ng/mL standard. From this solution, 25 μL was taken and mixed with 605 μL diluent buffer to make a standard solution of 4000 pg/mL. Then, the dilution series of standard solution were made, ranging from 16.38 to 4000 pg/mL. For analysis of the TGF β-1 on serum, 100 uL of sample was added to the 96-wells antibody-coated human TGF β-1 microplates (Merck, USA). The plate was covered and incubated for 2.5 hours at room temperature with gentle shaking. After incubation, the solution on the plate was discarded and washed four times using 300 uL of wash buffer. After the last wash, the remaining buffer on the plate was drained using clean paper towel. Then, the detection antibody solution (100 uL) was added to each well. The plate was covered and incubated for 1 hour at room temperature with gentle shaking. After incubation, the solution was discarded and washed with the same step previously mentioned for washing and draining. Afterwards, 100 uL of Streptavidin solution was added to each well, covered the plate, and incubated again for 45 minutes at room temperature with gentle shaking. After incubation, the solution was discarded and washed with the same step previously mentioned for washing and draining. Unto each well of the drained plate, 100 uL TMB One-Step Substrate Reagent was added, covered, and incubated for 30 minutes at room temperature in dark condition with gentle shaking. Then, 50 uL of stop solution was added to each well and the absorbance at 450 nm was measured using microplate reader (Tecan Infinite M200, Tecan, USA). The concentration of TGF β-1 on serum was obtained by interpolating the data from the standard curve.

### TNF-α measurement

Human TNF-α serum analysis from stunted and normal children sample, was conducted according to the manufacturer’s procedure (Proteintech, Wuhan, China). Antibody specific for TNF-α was precoated onto ninety-six-microwell, serum was diluted and added to the microplate and incubated for 60 min at 37°C. Plates were washed four times, and incubated with HRP-conjugated antibody. The color intensity which is proportional to the quantity of bound protein was measured at 450nm with the correction wavelength set at 630 nm.

### Lipopolysaccharide Binding Protein (LBP) assay

The measurement of lipopolysaccharide binding protein (LBP) on serum was conducted following the protocol mentioned on the human LBP ELISA kit protocol (Hycult Biotech, Uden, NL). Prior to the analysis, all reagents and buffer were brought to room temperature. For the preparation of LBP standard solution, around 450 uL was pipetted into the tube and diluted to make a standard solution ranging from 33.3 to 0 ng/mL. Before use, the microtiter plate was washed with 200 μL dilution buffer and hold for 20 second. After, about 100 μL of sample was pipetted into appropriate wells. The plate was covered and incubated for 1 hour at room temperature. After incubation, each well was washed like previously mentioned. Then, 100 μL of diluted biotinylated antibody was added to the wells. The plate was covered and incubated for 1 hour at room temperature. After incubation, each well was washed following the procedure previously mentioned. The procedure was then continued with the addition of 100 μL of diluted streptavidin-peroxidase to each well. The plate was covered and incubated for 1 hour at room temperature. After incubation, each well was washed like previously mentioned. Then, 100 μL of TMB substrate was pipetted to each well. The plate was covered and incubated for 30 minutes at room temperature. Afterwards, the TMB reaction was stopped by adding 100 μ5L of stop solution, and the absorbance at 450 nm was measured. The concentration of LBP on serum was obtained by interpolating the data from the standard curve.

### sIgA measurement

The method used for saline extraction of fecal immunoglobulins was adapted from that used by Ferguson et al. [16] essentially as described in Kusumo et al. [17]. In brief, approximately 1 g (wet weight) of feces was added to 10 ml extraction buffer (0.01 M phosphate-buffered saline [PBS] [pH 7.4], 0.5% Tween [Sigma-Aldrich, Poole, UK], and 0.05% sodium azide) and homogenized by vortexing. The homogeneous mixture were centrifuged at 1,500 x g for 20 min at 5°C. A portion of the supernatant (2 ml) was transferred to a sterile Eppendorf tube containing 20 μl of protease inhibitor cocktail (Sigma-Aldrich), and the tube was briefly vortexed to mix the contents. Samples were centrifuged at 10,000 x g for 10 min in a microcentrifuge, and the supernatants were transferred to clean Eppendorf tubes and stored at 20°C [16].

ELISA was performed for sIgA according to manufacturer instructions (Abbkine Scientific Co., Ltd, Wuhan, China). In brief, 100 μL standard, control and samples were added to the wells of a microtiter plate and incubated for 1 hour at room temperature on a horizontal mixer with shaking. Wells were subsequently aspirated and washed 5 times with 250 μl ELISA wash buffer (Abbkine) and then 100 μl peroxidase-labeled human anti-sIgA was added and the plate was incubated for 1 hour at room temperature with shaking on the horizontal mixer. The plate was washed 5 times as described above. After that 100 μl TMB substrate was added, and the plate was incubated for 10–20 minutes at room temperature. Next 50 μl ELISA STOP solution was added and mixed shortly. Absorption at 450 nm was measured with a plate reader reader [17]. Determination of human sIgA in feces was conducted by interpolating the data from the standard curve provided with the human sIgA ELISA Kit (Abbkine ranging from 32 to 1 μg/mL.

### SCFA measurement

#### Stool collection and extraction

Faecal samples were collected as described before [16]. In brief, faecal samples were collected in a stool container, kept in a cool box, and then stored in a -20°C freezer upon wrrival in the lab until being extracted. Faeces aliquots of 1 g of dry weight each, were transferred into a 25 mL plastic vial, containing 3 ml ethyl acetate and 3 ml formic acid, homogenized with a vortex and then centrifuged for 10 minutes at 3000 g. The supernatant (organic phases) was separated and transferred into a 15 mL plastic vial to which Na_2_SO_4_ anhydrate had been added. The samples were stored at −20°C until analysis with Gas chromatography–mass spectrometry (GC-MS).

#### GC-MS analysis

SCFA concentrations in the samples were determined using an Agilent Technology 6890 Gas Chromatograph with auto samplers and 5973 Mass Selective Detection and Chemstation Data System (Agilent, Singapore) and equipped with an HP-Innowax capillary column (30 m length; 0.25 mm internal diameter, with a 0.25 μm film thickness; Agilent). Samples (5 μl) were directly injected into the gas chromatograph using He as gas carrier at a constant flow rate of 0.8 ml/min. The temperature of the injector was kept at 230°C, and the split ratio was 8:1. Chromatographic conditions were as follows: initial oven temperature of 80°C, 8°C/min increase up to 220°C, 12 min constant at 220°C, and a ramp of 20°C/min increase up to 230°C to clean the column. In the MS detector, the electron impact energy was set at 70 eV. The data were collected as described in Kusumo et al. [18].

### Statistical methods

Differences between groups were investigated using the non-parametric Kruskal Wallis test. After the Kruskal–Wallis test, the Benjamini–Hochberg false discovery rate (FDR) was applied to correct for multiple comparisons. The non-parametric Spearman’s rank-order correlations were obtained between parameters and continuous variables. All of these calculation were performed by using the software package R (3.5.3) (R Core Team, http://www.R-project.org/) in RStudio. *Q*-values (adjusted *p*-values after FDR) were considered significantly different at *q* < 0.20.

## Results

### Correlation between immune parameters and microbial metabolites, and stunting

When immune parameters were compared between stunted children and children with normal nutritional status, only TGF-β was significantly higher in the stunted children (1782 ± 250 vs. 1661 ± 283 pg/ml, *q* < 0.2; Table 1). IL-10, TNFα, LPS binding protein (LBP) and secretory IgA (sIgA) did not differ between the groups.

**Table 1:** Parameters that showed significance between stunted and normal children (*q* < 0.2).

	p-value	q-value
TGF	0.009	0.113
valerate	0.017	0.130
SCFA	0.017	0.130
acetate	0.041	0.195

For all the microbial metabolites measured (acetate, propionate, butyrate, valerate, iso-butyrate, iso-valerate, total SCFA [acetate + propionate + butyrate + valerate] and total BCFA [iso-butyrate + iso-valerate]), concentrations were higher in feces of stunted children (Table 2). But after FDR correction for multiple comparison this was only significant for acetate, valerate and total SCFA (*q* < 0.2; Table 1).

**Table 2:** Average (± SD) concentrations of individual and total SCFA and BCFA.

	normal	stunted
acetate*	3.57 ± 3.87	6.28 ± 6.42
**propionate**	2.36 ± 4.73	4.27 ± 10.11
***n*-butyrate**	1.67 ± 2.01	2.62 ± 3.47
valerate*	0.38 ± 0.44	0.87 ± 1.83
SCFA*	7.97 ± 9.72	14.03 ± 17.48
**iso-butyrate**	0.13 ± 0.19	0.37 ± 0.76
**iso-valerate**	0.25 ± 0.63	0.70 ± 1.56
**BCFA**	0.39 ± 0.81	1.07 ± 1.94

* *q* < 0.2.

### Correlation between immune parameters and microbial metabolites, and gut microbiota composition

**Fig 2:**
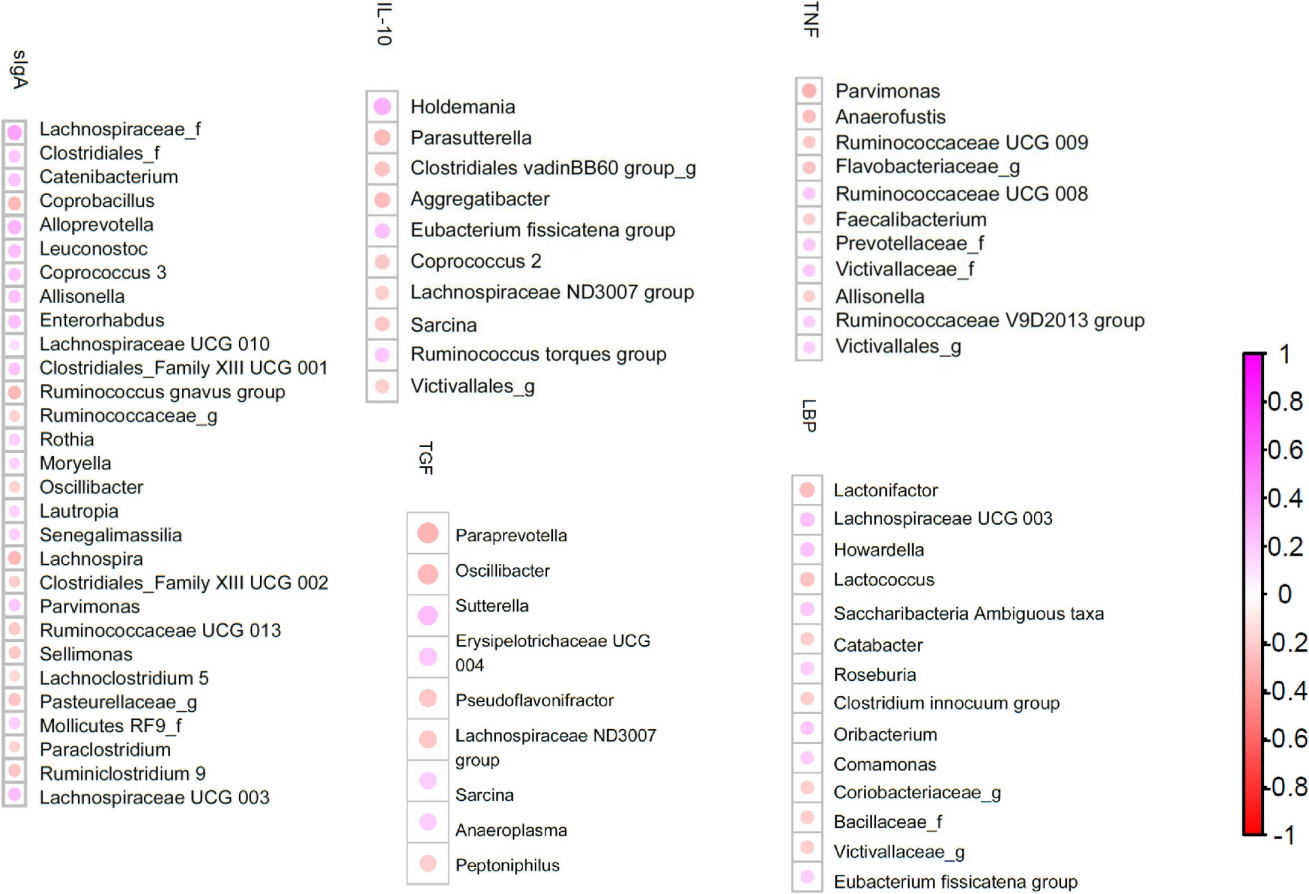
Spearman correlation between immune parameters and gut microbiota composition. Only taxa that are significant (q < 0.2) are shown, ranked from highest *q* to lowest *q*. Purple: Positive Spearman correlation factor (rho); red: Negative rho.

**Fig 3:**
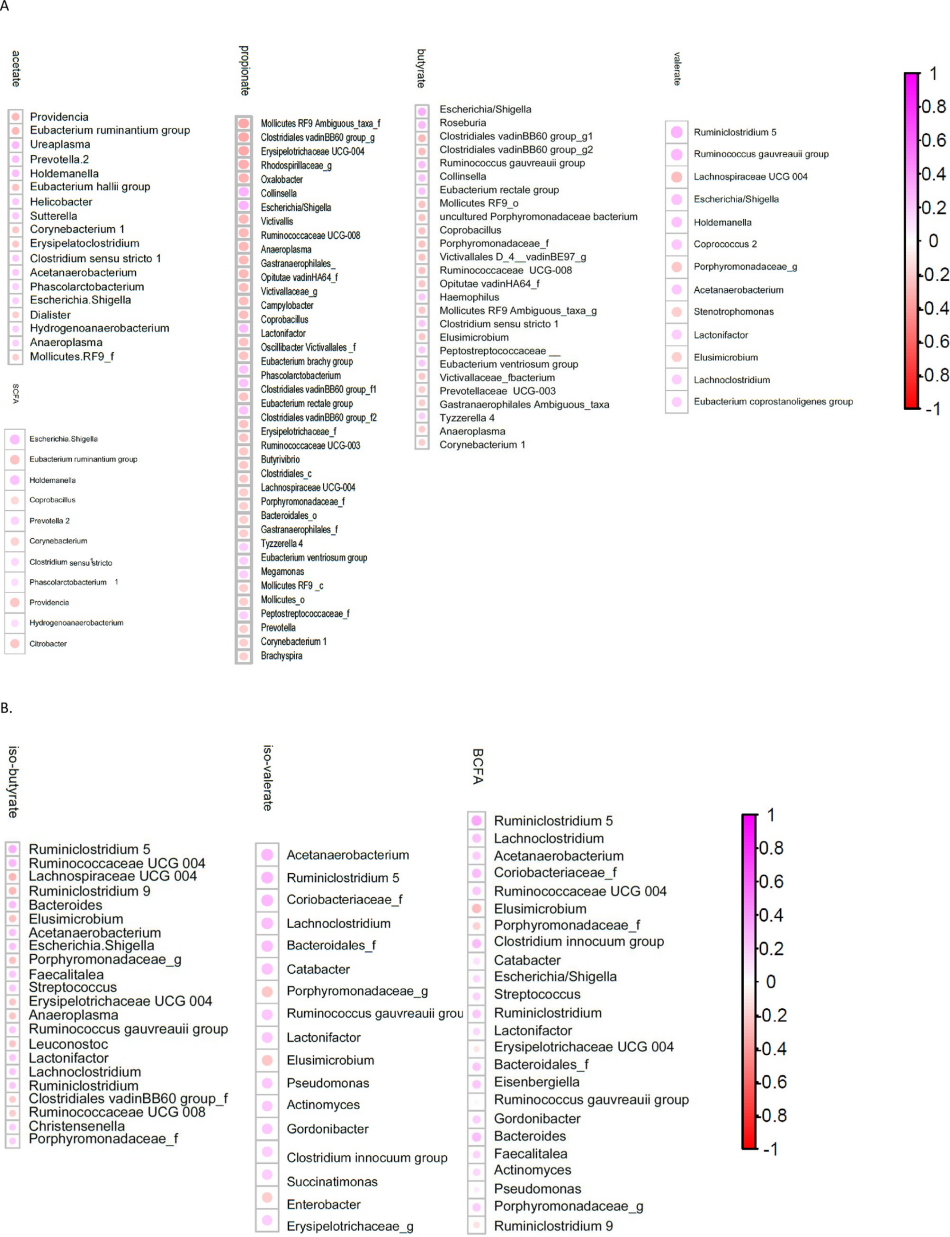
Spearman correlation between microbial metabolites and gut microbiota composition. Only taxa that are significant (q < 0.2) are shown, ranked from highest *q* to lowest *q*. A) individual and total SCFA; B) individual and total BCFA. Purple: Positive Spearman correlation factor (rho); red: Negative rho.

There were several correlations between immune parameters and gut microbiota composition (Fig 2). Similarly, the microbial metabolites correlated with gut microbiota composition (Fig 3).

### Correlation between macro- and micronutrient intake and stunting and gut microbiota composition

As expected, at the macronutrient level, intake for carbohydrate, fat, protein and energy was lower in stunted children than in children with normal nutritional status (Table 3; significant for fat and energy intake).

**Table 3:** Average (± SD) of macronutrients, energy and dietary fibre intake.

	normal	stunted
energy (kcal)*	1161 ± 430	1011 ± 490
protein (g)	34.91 ± 15.00	34.59 ± 28.77
fat (g) *	38.19 ± 18.70	32.24 ± 18.47
carbohydrate (g)	165.15 ± 70.79	144.59 ± 72.07
dietary fiber (g)	5.01 ± 3.13	4.30 ± 2.28

* significantly different (*q* < 0.2) between stunted children and children of normal nutritional status.

Table 4 shows the correlation between microbial taxa and macronutrients. Several taxa are correlated with multiple macronutrients. E.g., *Eisenbergiella* is positively correlated with carbohydrate, fat and protein (and hence with energy). Similarly, a genus within the Succinivibrionaceae family, belonging to the Gamma-proteobacteria, is correlated with carbohydrate, fat, protein and energy, but negatively in all cases. Several taxa that negatively associate with carbohydrate intake, also negatively associate with energy and/or dietary fibre intake: an uncharacterized genus in the Victivallaceae family, an uncharacterized family in the Victivalles order, an uncharacterized genus in the Clostridiales vadinBB60 group, an uncharacterized genus in the Erysipelotrichaceae family, and the genus *Erysipelotrichaceae* UCG-004. The presence of the methane-producing *Gelria* was positively correlated with energy, protein and fat intake. The genus *Lachnospiraceae* ND3007 group positively correlates with energy, protein and fat intake. The genus *Eubacterium xylanophilum* group of the Lachnospiraceae positively correlates with protein and fat intake. The *Prevotella* taxon, present at relatively low abundance (0.0022%) compared to the highly abundant *Prevotella* 9 (on average 27%) was negatively associated with energy, carbohydrate and protein intake.

**Table 4:** Correlation between nutrient and energy intake and microbial taxa. Only taxa that are significant (q < 0.2) are shown.

	energy			carbohydrate			protein			fat			dietary fibre		
taxa	p-value	q-value	rho-value	p-value	q-value	rho-value	p-value	q-value	rho-value	p-value	q-value	rho-value	p-value	q-value	rho-value
Eisenbergiella	0.029	0.144	0.19	0.033	0.157	0.19	0.011	0.055	0.22	0.019	0.096	0.21			
Succinivibrionaceae_g	0.013	0.069	-0.22	0.042	0.198	-0.18	0.003	0.014	-0.26	0.029	0.144	-0.19			
Victivallaceae_g	0.036	0.167	-0.18	0.013	0.066	-0.22							0.036	0.171	-0.18
Victivallales_f	0.020	0.099	-0.20	0.038	0.180	-0.18	0.031	0.148	-0.19						
Clostridiales vadinBB60 group_g	0.035	0.167	-0.18	0.011	0.056	-0.22							0.032	0.157	-0.19
Erysipelotrichacea_g	0.016	0.081	-0.21	0.005	0.027	-0.24							0.013	0.068	-0.22
Erysipelotrichaceae UCG-004				0.009	0.047	-0.23							0.016	0.084	-0.21
Prevotella	0.014	0.070	-0.22	0.025	0.125	-0.20				0.040	0.194	-0.18			
Candidatus_Stoquefichus	0.039	0.178	0.18				0.033	0.157	0.19						
Eubacterium xylanophilum group							0.036	0.167	0.18	0.037	0.181	0.18			
Clostridiales_f	0.031	0.149	0.19				0.013	0.067	0.22						
Lachnospiraceae ND3007 group	0.006	0.034	0.24				0.026	0.126	0.20	0.035	0.174	0.18			
Gelria	0.036	0.167	0.18				0.036	0.167	0.18	0.037	0.181	0.18			
Lachnoclostridium	0.035	0.166	0.19	0.032	0.154	0.19	0.011	0.057	0.22						
Porphyromonas	0.041	0.188	-0.18	0.042	0.198	-0.18									
Stenotrophomonas	0.021	0.105	0.20	0.028	0.139	0.19									
Veillonellaceae_f							0.026	0.126	0.20				0.021	0.106	0.20
Peptococcus							0.033	0.157	0.19	0.018	0.096	0.21			
Ruminiclostridium 5				0.023	0.116	0.20									
Succinatimonas							0.022	0.108	0.20						
Anaerofilum										0.026	0.132	-0.20			
Corynebacterium 1										0.038	0.187	-0.18			
Methanosphaera										0.012	0.066	-0.22			
Bacteroidales S24-7 group_g													0.031	0.153	0.19
Carnobacteriaceae_f													0.028	0.138	0.19
Lachnospiraceae UCG-008													0.019	0.096	-0.21
Rothia													0.030	0.147	-0.19

Parents or guardians recorded 23 different food items on the 24 h recall food questionnaire. These correlated mostly with taxa that were present only in a few children, although in a few cases the more abundant taxa showed correlation with food item intake (S1 Table).

## Discussion

We expected the stunted children to have higher inflammatory plasma cytokines, and lower IL-10 and TGF, as it has been shown that in low-income regions with poor sanitation, undernutrition is frequently associated with chronic intestinal inflammation [19]. Instead, we showed that TGF was significantly higher in stunted children, and there were no differences in TNF-alpha, LBP (as marker for (low-grade) inflammation caused by LPS), nor in IL-10, while also fecal secretory IgA was not significantly different. Secretory IgA is known to mediate host-microbial homeostasis in the intestine [20], and acutely undernourished children have been shown to have altered IgA recognition of the fecal microbiota [20]. This was however not reflected in altered concentrations in our study.

For all microbial metabolites, the concentrations were higher in feces of stunted children than in that of children with normal nutritional status (significant for acetate, valerate and total SCFA, Tables 1 and 2). SCFA have been shown to be immunomodulatory [21,22]. As we show an overall higher concentration of individual (and total) SCFA concentration in feces of the stunted children, it could be that these molecules are responsible for an anti-inflammatory response in the stunted children, although the increased excretion of SCFA also leads to energy-loss as we discussed in our previous report on the gut microbiota profile of these children [13].

As expected, at the macronutrient level, intake for carbohydrate, fat, protein and energy was lower in stunted children than in children with normal nutritional status (Table 3). In our previous report, we calculated dietary fibre intake as well, which was also lower in stunted children (Table 3), although not significantly [13]. In the stunted children we reported a significant lower relative abundance (RA) of *Prevotella* 9 [13]. *Prevotella* has been correlated to dietary fibre intake [23], and hence the lower RA could be caused by the lower dietary fibre intake of the stunted children, although it should be mentioned that the food intake reported here, was based on a 24 h food recall. Therefore, we cannot exclude that the reduced RA of *Prevotella* is caused by other reasons, also prior to the previous 24 h. Nevertheless, it is not unreasonable to assume that if the stunted children ate less dietary fibre in the past 24 h, that they may also have done so in the period before that, and perhaps throughout their entire life.

When the microbial taxa were correlated to macronutrients, several taxa (discussed bel0w) were correlated with multiple macronutrients (Table 4). *Eisenbergiella* is a member of the family Lachnospiraceae. There is very limited biological function information on this genus, and the implication of *Eisenbergiella* in human health has yet to be defined. *Eisenbergiella massiliensis* has been collected from stools of individuals undergoing bariatric surgery [24]. *Eisenbergiella tayi* has been isolated from human blood [25].

A genus within the Succinivibrionaceae family negatively correlated with carbohydrate, fat, protein and energy. In a recent study, Succinivibrionaceae correlated with lean individuals [26], and hence could be negatively associated with energy intake as we show here.

*Erysipelotrichaceae* UCG-004 is negatively correlated with carbohydrate and dietary fibre intake. The role of Erysipelotrichaceae is unclear, and four different lineages within Erysipelotrichaceae have been identified that respond differently to diet or host health phenotypes [27].

The presence of the methane-producing *Gelria*, that here was positively correlated with energy, protein and fat intake, has been positively associated with functional constipation [28]. In this study we only scored the occurrence of diarrhoea as a measure for hygienic status, but we did not look at stool consistency or constipation.

The genus *Lachnospiraceae* ND3007 group positively correlates with energy, protein and fat intake. The role of Lachnospiraceae in health and disease is controversial [29]. Lachnospiraceae have been described to belong to the core of gut microbiota, colonizing the host from birth and increasing during the host’s life. Members of Lachnospiraceae are among the main producers of the benefiial SCFA, but at the same time different taxa of Lachnospiraceae have also been associated with different diseases, both in the gut as well as systemically [29]. Their impact on the host physiology is often inconsistent across different studies.

The genus *Eubacterium xylanophilum* group of the Lachnospiraceae positively correlates with protein and fat intake. In a recent study in mice fed a high-fat diet (HFD), the abundance of this genus was higher in the HFD fed mice than in the controls [30]. On the other hand, in an *in vitro* study where wheat bran was fed to a human microbial community, this genus was highly enriched [31], in agreement with the fact that Lachnospiraceae are known to be involved in complex carbohydrate fermentation and SCFA production. However, in our study there was no significant correlation with dietary fibre intake, likely because the intake of complex cereal fibres was low.

The overall genus *Prevotella* has been correlated with dietary fibre intake [23], which was not observed here. The biological relevance of the negative association shown here between the *Prevotella* taxon and energy, carbohydrate and protein intake is questionable as this *Prevotella* taxon was present in relatively few children and at low abundance. Moreover, the correlation between the overall genus *Prevotella* and dietary fibre intake has been observed in adults, and it remains to be seen whether this is also the case for children between 3 and 5 years of age.

In conclusion, the correlations observed with immune parameters, macronutrients and microbial metabolites may provide indications on how to modulate the gut microbiota of stunted children such that their growth lag can be corrected. Our current efforts are targeted towards an intervention strategy with (local) functional food components in an even larger population of Indonesian stunted children.

## Supporting information

S1 Checklist Trend checklist. (DOCX)

S1 Table Correlations between major OTUs and intake of several recorded food items. Values are the significant q-values (FDR-corrected p-values < 0.2) of a Kruskal-Wallis comparison between children that did ingest the food-item and those that did not. Values in bold show taxa that are higher in children that ate the food item, values in italic show taxa that are lower in children that ate the food item.
(XLSX)

S1 File Study protocol in English. (PDF)

S2 File Study protocol in original Bahasa Indonesia language. (PDF)

S1 Data (CSV)
